# Association between estimated glucose disposal rate and metabolic syndrome in older adults with sarcopenia

**DOI:** 10.3389/fnut.2026.1795633

**Published:** 2026-06-29

**Authors:** Min Zou, Hui Qin, Qing Xia, Jian Kang

**Affiliations:** 1 The Department of Nutrition, 921 Hospital of the Joint Support Force of the People's Liberation Army of China, Changsha, China; 2 The Laboratory Department, 921 Hospital of the Joint Support Force of the People's Liberation Army of China, Changsha, China; 3 The Disease Prevention and Control Department, 921 Hospital of the Joint Support Force of the People's Liberation Army of China, Changsha, China

**Keywords:** eDGR, gucose disposal rate, machine learning, metabolic syndrome, sarcopenia

## Abstract

**Objective:**

Sarcopenia, an age-related syndrome marked by muscle mass and function decline, is closely linked to various adverse clinical outcomes. Insulin resistance (IR) has emerged as a pivotal link between sarcopenia and metabolic syndrome (MetS), significantly influencing their pathophysiological interactions. This study aimed to investigate the potential correlation between estimated glucose disposal rate (eGDR) and MetS among patients with sarcopenia, offering new insights into stratified prevention and treatment strategies.

**Methods:**

A total of 970 elderly individuals who visited the 921 Hospital of the Joint Logistics Support Force of the Chinese People's Liberation Army from January 2023 to December 2024 were enrolled. Participants were categorized into three groups, including the non-sarcopenia group (*n* = 613), the sarcopenia group (*n* = 307) and the sarcopenia with MetS group (*n* = 50). Key variables were selected using univariate screening, the support vector machine recursive feature elimination (SVM-RFE) algorithm, and the Boruta algorithm. Three logistic regression models were constructed to evaluate the association between eGDR and MetS.

**Result:**

The prevalence of MetS in elderly individuals with sarcopenia was 14.01% (50/357), significantly higher than that of those without sarcopenia (3.10%, 19/613), with an odds ratio (OR) of 5.09 (*95% CI*: 2.95–8.79). Among the 357 sarcopenia patients, the average eGDR level in the sarcopenia with MetS group was 9.60 (9.21, 10.03) mg/kg/min, which was lower than that in the sarcopenia group, 10.82 (10.55, 11.09) mg/kg/min (*Z* = 2.64, *P* < 0.01). The Boruta algorithm identified crucial variables for developing a logistic regression model, indicating an OR for eGDR of 0.54 (95% CI: 0.30–0.84). The ORs for frailty and elevated cholesterol levels were 1.47 (95% CI: 1.01–2.94) and 3.94 (95% CI: 2.47–6.69), respectively. Model performance comparison showed that the Boruta and SVM-RFE-based models had higher area under the ROC curve values (0.78 and 0.75, respectively) than the univariate screening model (*P* < 0.05). Furthermore, subgroup analysis confirmed the robustness of the association between eGDR and MetS in elderly patients with sarcopenia.

**Conclusion:**

In the older adult group, patients with sarcopenia have a higher possibility of developing metabolic syndrome (MetS) compared with those without sarcopenia. In addition to lower eGDR levels, frailty, elevated uric acid, BMI, and MDA levels are associate with a higher prevalence of MetS in individuals with sarcopenia. Machine learning-based regression models outperform univariate screening models in predicting MetS risk.

## 1 Introduction

As China's demographic shift toward an aging society becomes more pronounced, sarcopenia, an age-related condition, has emerged as a significant public health concern, affecting the health outcomes of the elderly population (1). Global epidemiological data suggest that the prevalence of sarcopenia among community-dwelling older adults ranges from approximately 10% to 27%, with projections suggesting that the number of individuals with sarcopenia worldwide will reach 500 million by the year 2050 (2). In China, the rising aging rate has heightened the public health implications of sarcopenia, and statistics reveal that the overall prevalence of sarcopenia among Chinese adults aged 65 and above is 16.5%, with a clear increasing trend observed as age advances (3). Recent research has highlighted that sarcopenia rarely occurs in isolation; it often coexists with other chronic diseases, encompassing cancer, COPD, heart failure, and kidney and liver diseases (4, 5). MetS, a cluster of clinical conditions centered around central obesity and IR, is not only prevalent among older adults but also closely associated with sarcopenia (6). These two conditions share a common pathophysiological basis, particularly IR, which elevates the risk of type 2 diabetes mellitus, cardiovascular disease, and cancer (7, 8). The bidirectional relationship between sarcopenia and chronic diseases—where each condition exacerbates the onset, progression, and adverse outcomes of the other—underscores the critical need for timely detection and evidence-based, multidomain interventions.

The growing availability of large-scale, high-quality clinical and multimodal datasets—coupled with advances in scalable, interpretable machine learning algorithms—has positioned machine learning as a pivotal tool for precision risk stratification and timely, personalized early intervention across a spectrum of chronic diseases (9, 10). Machine learning models are uniquely suited to modeling complex, nonlinear relationships and integrating high-dimensional, heterogeneous feature spaces—without imposing restrictive parametric assumptions about variable interactions—making them particularly powerful for both predictive risk modeling and hypothesis-generating discovery of associations between chronic diseases and multifactorial clinical, behavioral, and biological determinants (11–13). Notably, Rong et al. (14) recently applied a multilayer perceptron (MLP) model to identify age, BMI, total cholesterol, and sex—as key discriminative features for sarcopenia risk stratification among patients with chronic diseases—achieving a robust ROC AUC of 0.912 in external validation. Xia et al. (15) demonstrated that ensemble machine learning models—including random survival forests (RSF) and gradient boosting survival (GBS)—achieve superior discrimination for overall survival prediction in pulmonary papillary adenocarcinoma, with statistically significant improvements in both C-index and time-dependent AUC over the conventional Cox proportional hazards model.

The hyperinsulinemic-euglycemic clamp (HEGC), acknowledged as the gold standard for evaluating whole-body insulin sensitivity, enables direct and quantitative measurement of glucose utilization under insulin stimulation (16). However, its complex procedure and high cost limit its widespread clinical use. eGDR, a composite parameter derived from routine clinical parameters such as waist circumference, blood pressure, and glycated hemoglobin, serves as a practical proxy for IR and eGDR has demonstrated predictive value for various diabetes-related outcomes, frailty, and cardiovascular disease risk (17–19). In Asian population-based cohort studies, skeletal muscle mass has been inversely correlated with eGDR (20). In spite of these insights, previous research has primarily focused on patients with diabetes or sarcopenia alone, resulting in notable gaps in our understanding of the strength, characteristics, and influencing factors of the association between eGDR and MetS in elderly individuals with sarcopenia. In order to address this gap, this study focuses on elderly individuals with sarcopenia. Using univariate screening alongside two feature selection algorithms—the SVM-RFE algorithm and the random forest-based Boruta algorithm—logistic regression models were constructed to analyze the association between eGDR levels and MetS, as well as its influencing factors. The study aims to provide an objective foundation for early intervention and stratified prevention and management strategies in patients with sarcopenia.

## 2 Materials and methods

### 2.1 Study design and population

This study enrolled 970 elderly individuals who visited the 921 Hospital of the Joint Support Force of the People's Liberation Army of China between December 31, 2023, and December 31, 2024. The inclusion criteria were: (1) age ≥ 60 years; (2) diagnosed with sarcopenia according to the 2019 diagnostic criteria of the Asian Working Group for Sarcopenia (AWGS) (16) (as detailed in Section 2.3); (3) no history of severe trauma or surgery within the past 2 weeks; (4) good compliance, willingness to participate in all required tests, and provision of written informed consent; the exclusion criteria were as follows: (1) a definite diagnosis of type 1 or type 2 DM; (2) serious liver and kidney dysfunction or end-stage heart failure (ALT and/or AST levels exceeding three times the upper limit of normal; eGFR < 30 mL/min/1.73 m^2^; New York Heart Association functional class III or IV); (3) presence of edema; (4) severe wasting illnesses, including but not limited to acute exacerbation of chronic obstructive pulmonary disease, uncontrolled thyroid disease, and active rheumatoid arthritis; (5) prolonged treatment (≥3 months) with high-dose glucocorticoids (e.g., prednisone equivalent dose ≥ 0.5 mg/kg/d) or immunosuppressant agents; (6) a serious mental or psychological disorder that makes it impossible to cooperate with the normal examination process.

### 2.2 Data collection

Information on patient demographics and laboratory test results was collected. Demographic data included gender, age, height, weight, waist circumference, calf circumference, and blood pressure. Laboratory test results covered fasting blood glucose, glycated serum protein, glycated hemoglobin, albumin, blood urea nitrogen (BUN), creatinine, cholesterol, triglycerides, high-density lipoprotein, high-sensitivity C-reactive protein, uric acid, superoxide dismutase (SOD) activity, MDA, 25-hydroxyvitamin D_3_, white blood cell count, as well as lymphocyte count, among other biochemical indicators.

### 2.3 Variable definitions

The diagnostic criteria for MetS were based on the Chinese Guidelines for Prevention and Treatment of Type 2 Diabetes (2017) issued by the Chinese Diabetes Society (CDS) (21), and these criteria include: (1) waist circumference ≥90 cm (men) or ≥85 cm (women); (2) fasting plasma glucose ≥6.1 mmol/L and/or 2-h postprandial plasma glucose ≥7.8 mmol/L, or a confirmed diagnosis of diabetes mellitus under treatment; (3) systolic blood pressure ≥130 mmHg and/or diastolic blood pressure ≥85 mmHg, or a confirmed diagnosis of hypertension under treatment; (4) fasting triglycerides ≥1.70 mmol/L; (5) fasting high-density lipoprotein cholesterol < 1.04 mmol/L. A diagnosis of MetS was established if patients met three or more of these criteria.

In this study, eGDR was used as an indicator of IR. The eGDR was calculated using the formula proposed by Roberts et al. (22), as follows: eGDR (mg/kg/min) = 21.158 – (0.09 × WC) – (3.407 × hypertension) – (0.551 × HbA1c). WC represents waist circumference (cm). Hypertension was coded as 1 for yes and 0 for no. HbA1c denotes glycated hemoglobin (%). Hypertension was defined as systolic blood pressure ≥ 140 mmHg and/or diastolic blood pressure ≥ 90 mmHg.

Sarcopenia was diagnosed using the cutoff values recommended by the AWGS, 2019 (23). Participants with low muscle mass, plus low muscle strength, and/or low physical performance were diagnosed with sarcopenia. In this study, bioelectrical impedance analysis (BIA) was utilized to assess muscle mass. Low muscle mass was defined as a skeletal muscle index (ASM/height^2^) of < 7.0 kg/m^2^ in men and < 5.7 kg/m^2^ in women, according to established criteria (ASM, Appendicular Skeletal Muscle). Grip strength was utilized to assess muscle strength loss in men and women, featuring low muscle strength defined as grip strength < 28 kg in men and < 18 kg in women, according to established criteria. Physical performance was assessed by the time required to complete five repeated chair stands, with a duration of ≥12 s showing reduced physical performance.

The frailty status of the participants was evaluated using the Frailty Screening Questionnaire (FSQ) (24), and this assessment included six domains: slow walking, reduced grip strength, weight loss, reduction in physical activity, cognitive impairment, and fatigue (total score: 12 points). A score of ≥6 points was defined as indicating frailty.

### 2.4 Statistical analysis

The statistical analyses were performed using IBM SPSS Statistics 25 to compare groups, and the Shapiro–Wilk test was used to assess the normality of continuous variables. Normally distributed continuous variables are presented as mean ± standard deviation, while non-normally distributed data are expressed as median (interquartile range). Group comparisons were conducted using one-way ANOVA or the Mann-Whitney U test. Categorical variables are reported as counts (percentages), and intergroup differences were assessed using the χ^2^ test or Fisher's exact test. All statistical tests were two-tailed, and a *P*-value < 0.05 was considered statistically significant.

During the model development phase, statistical analyses were carried out using Python 3.11 and R software version 4.3 with relevant packages. Three distinct approaches were used to construct logistic regression models. For variable screening, univariate analysis was used to select variables with a *P*-value < 0.05 in intergroup comparisons as candidate features, and machine learning algorithms were applied for feature selection: the SVM-RFE algorithm and the random forest–based Boruta algorithm were implemented to identify the top 5 feature variables in terms of importance from the variable pool to construct the optimal feature subset. Based on the results of feature screening, logistic regression models were fitted using a forward-backward stepwise selection method. To be specific, the SVM-RFE algorithm from the scikit-learn library was used with a linear kernel support vector machine. The RFECV function was employed to automatically determine the best feature subset, with key parameters set as follows: cv = 5, scoring = ‘roc_auc', min_features_to_select = 5, and step = 1. The Boruta algorithm was executed using the Boruta package in R with maxRuns = 500 and ntree = 1,000. Meanwhile, internal validation was conducted for the models constructed by the above three strategies respectively. Internal validation was performed using 1000-bootstrap resampling, and the optimism-corrected AUC was computed.

Lastly, multicollinearity diagnostics were carried out by calculating the variance inflation factor (VIF) for each variable included in the predictive models, with a VIF > 5 indicating significant multicollinearity. Non-linear dose-response relationships between eGDRs and the outcomes were modeled using restricted cubic splines (RCS). To compare model performance, we calculated the AUC, a metric for prediction accuracy, and utilized the DeLong test to determine the statistical significance of any observed differences. Subgroup analyses and leave-one-out (LOO) approach and E-value sensitivity analysis were employed to evaluate the robustness of the findings. Subgroup analyses were conducted stratified by potential confounders (gender, age, BMI and WHtR) that might influence the results. In LOO sensitivity analysis, we sequentially omitted WC and blood pressure components from the eGDR formula (set the coefficient of the excluded variable to 0), then recomputed the eGDR score and refitted the regression model, to assess the individual contribution of WC and blood pressure to the overall association.

## 3 Results

### 3.1 Information description

A total of 970 individuals were included in this study, encompassing 461 men (47.5%) and 509 women (52.47%), and the average age of participants was 84.66 ± 11.45 years. Among them, 357 individuals were diagnosed with sarcopenia, showing a prevalence rate of 36.80% (357/970). Patients with sarcopenia exhibited lower ASM (5.78 ± 1.23 kg/m^2^) and grip strength (18.42 ± 8.38 kg), as well as a longer FTSTS time (14.64 ± 3.53 s), compared to those without sarcopenia. See Table 1. MetS was identified in 69 participants, accounting for a prevalence of 7.11% (69/970). Among the 357 individuals with sarcopenia, 50 were diagnosed with MetS, resulting in a prevalence of 14.01% (50/357); in contrast, among the 613 individuals without sarcopenia, the prevalence of MetS was significantly lower at 3.10% (19/613), compared to the sarcopenic group (χ^2^ = 40.61, *P* < 0.01). See Table 2.

**Table 1:** Comparison of baseline characteristics between sarcopenia group (*n* = 357) and non-sarcopenia group (*n* = 613).

Group	Count	Age (years)	Male (n, %)	ASM/height^2^ (kg/m^2^)	Grip strength (kg)	FTSTS (s)
Sarcopenia	357	83.88 ± 6.27	174 (48.74%)	5.78 ± 1.23	18.42 ± 8.38	14.64 ± 3.53
Non-Sarcopenia	613	85.05 ± 14.51	297 (48.45%)	7.71 ± 3.46	23.57 ± 9.37	9.73 ± 4.47
Statistical test values		1.74	0.01	12.52	8.83	18.90
*P-*value		0.08	0.89	< 0.01	< 0.01	< 0.01

Data are presented as mean ± SD or *n* (%). ASM represents Appendicular Skeletal Muscle Mass. FTSTS represents Five Times Sit to Stand Performance.

**Table 2:** Comparison of the prevalence of MetS between the sarcopenia group (*n* = 357) and the non-sarcopenia group (*n* = 613).

Group	MetS	Non-MetS	Prevalence(%)	χ^2^	*P-*value	OR (95% CI)
Sarcopenia	50	307	14.01	40.61	< 0.01	5.09 (2.95~8.79)
Non-Sarcopenia	19	594	3.10			
Total	69	901	\			

### 3.2 Comparative analysis between sarcopenic patients and sarcopenic patients with MetS

Among the 357 participants with sarcopenia, further comparative analyses were conducted by dividing them into two groups based on the presence or absence of MetS: the sarcopenia group (*n* = 307) and the sarcopenia with MetS group (*n* = 50). The results revealed that the sarcopenia with MetS group exhibited a higher prevalence of frailty and a lower eGDR compared to the sarcopenia group. To be specific, the frailty prevalence was 66.00% (33/50) in the sarcopenia with MetS group, while it was 50.81% (156/307) in the sarcopenia group. The difference in frailty prevalence between the two groups was statistically significant (χ^2^ = 4.58, *P* = 0.03).

The eGDR in the sarcopenia group was 10.82 (10.55, 11.09) mg/kg/min, which was notably higher than that in the sarcopenia with MetS group [9.60 (9.21, 10.03) mg/kg/min; *Z* = 2.64, *P* < 0.01). In terms of age, gender, BMI, calf circumference, and waist-to-height ratio (WHtR), no statistically significant differences were observed between the two groups, as all *P*-values exceeded 0.05 (Table 3).

**Table 3:** Comparison of baseline characteristics between the sarcopenia group (*n* = 307) and the sarcopenia combined with MetS group (*n* = 50).

Group	Count	Age	Gender, *n* (%)		BMI (kg/m^2^)	Calf circumference (cm)	WHtR
			Male	Female			
Sarcopenia	307	84.04 ± 11.01	152 (48.86)	155 (50.49)	20.04 ± 2.64	32.13 ± 3.37	0.47 ± 0.05
Sarcopenia and MetS	50	81.82 ± 9.51	22 (44.00)	28 (56.00)	20.41 ± 1.96	32.62 ± 2.12	0.49 ± 0.07
Statistical test values		3.28	0.53		0.86	1.00	1.21
*P-*value		0.07	0.47	0.35		b0.32	0.27

**Figure 1:**
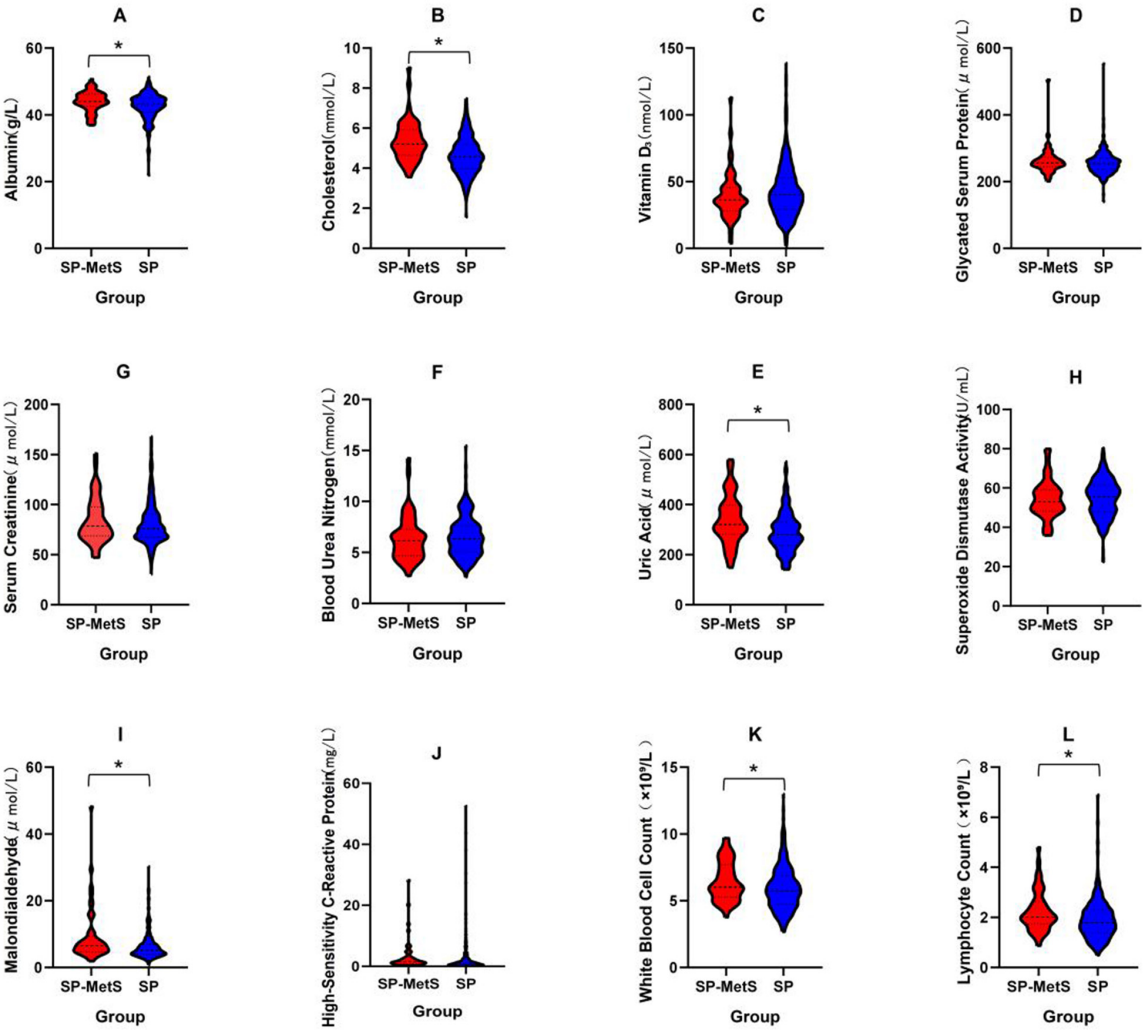
Comparison of Laboratory Indicators Between the Sarcopenia Group (*n* = 307) and the Sarcopenia Combined with MetS Group (*n* = 50). SP-MetS denotes the sarcopenia combined with MetS group; SP denotes the sarcopenia group; *indicates a statistically significant difference between groups.

When comparing nutrition-related indices between the two groups, it was found that the sarcopenia with MetS group had higher levels of cholesterol and albumin compared to the sarcopenia group. In patients with sarcopenia combined with MetS, the albumin level was 43.96 ± 3.08 g/L and the cholesterol level was 5.36 ± 1.02 mmol/L, while in those with sarcopenia alone, albumin was 42.51 ± 3.96 g/L and cholesterol was 4.63 ± 0.92 mmol/L, both of which were significantly lower than in the former group (*P* < 0.01). No significant differences were observed between the two groups regarding glycated serum protein or 25-hydroxyvitamin D_3_ levels see Figure 1.

Patients with sarcopenia combined with MetS also exhibited higher levels of oxidative stress markers and inflammatory factors compared to those with sarcopenia alone. The MDA level in the sarcopenia with MetS group was 9.16 ± 8.06 μmol/L, significantly higher than the 6.97 ± 3.88 μmol/L observed in the sarcopenia group (*F* = 23.30, *P* < 0.01). In the meantime, the white blood cell count and lymphocyte count in the sarcopenia with MetS group were 6.45 × 10?/L and 2.23 × 10?/L, respectively, which were also elevated compared to the sarcopenia group (5.91 × 10?/L and 1.89 × 10?/L). Uric acid levels were significantly higher in the sarcopenia with MetS group (341.94 ± 9.93 μmol/L) than in the sarcopenia group (290.27 ± 7.98 μmol/L), with a significant difference between the two groups (*F* = 27.86, *P* < 0.01). However, no statistically significant differences were found between the two groups for serum creatinine, BUN, SOD activity, or high-sensitivity C-reactive protein (hs-CRP) levels see Figure 1.

### 3.3 Association between glucose disposal rate and mets in patients with sarcopenia

Among the 357 patients diagnosed with sarcopenia, we developed logistic regression models using three distinct variable selection methods: univariate screening, the Boruta algorithm, and the SVM-RFE algorithm. The occurrence of MetS in elderly sarcopenic patients served as the dependent variable (coded as: yes = 1, no = 0). In all three models, eGDR showed a significant link with MetS. Based on the comparison of baseline characteristics and laboratory test indicators, variables like albumin, cholesterol, uric acid, MDA, white blood cell count, lymphocyte count, and eGDR were included in the multivariate logistic regression model. The results indicated that the OR for glucose disposal rate was 0.64 (95% CI: 0.38–0.98), suggesting that a higher eGDR is linked to a lower prevalence of MetS in patients with sarcopenia; conversely, elevated uric acid levels (OR = 1.01; 95% CI: 1.01–1.02), and high cholesterol levels (OR = 3.49, 95% CI: 3.30–5.26) were significantly related to an increased prevalence of MetS.

**Figure 2:**
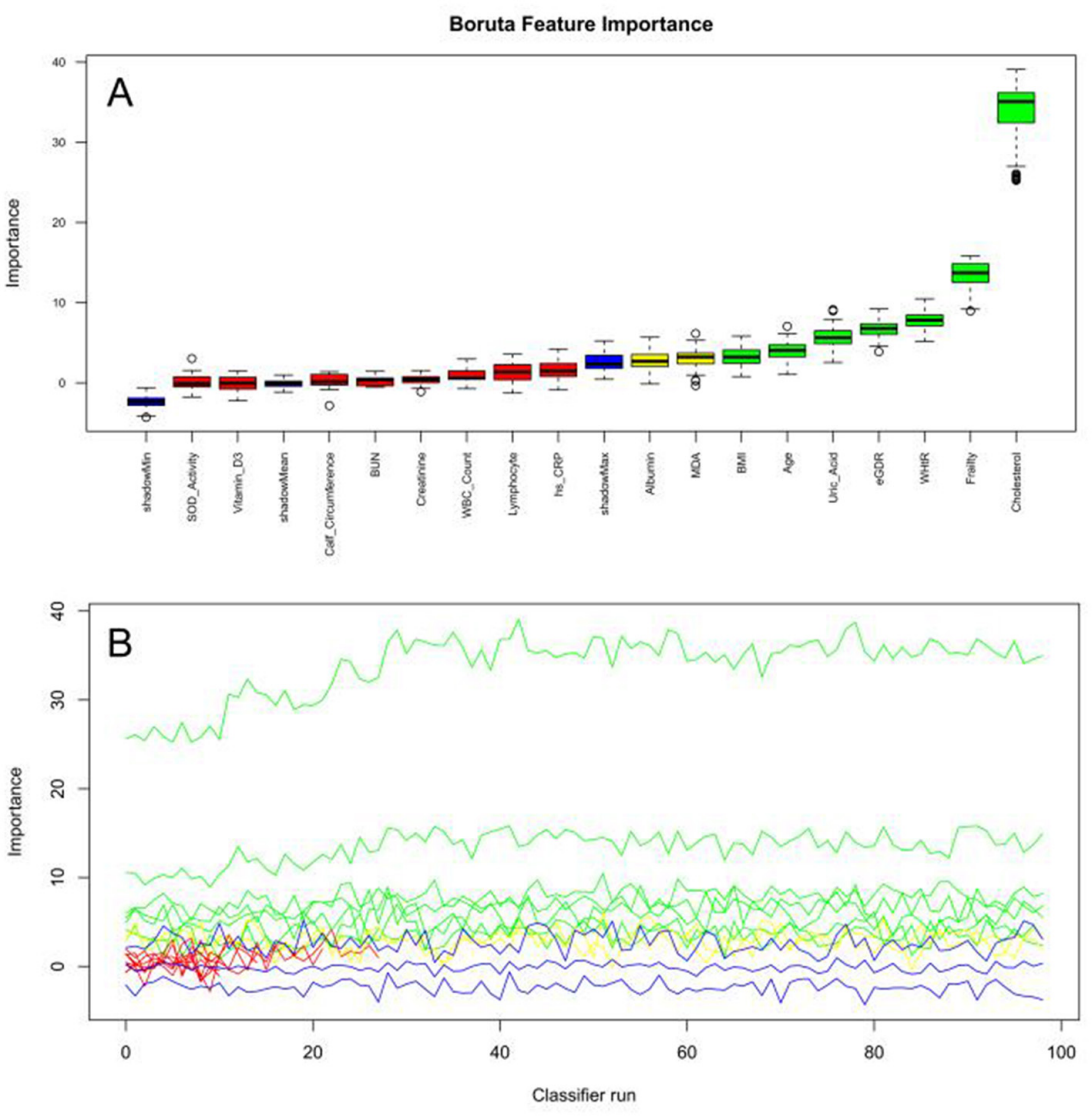
Feature subset selected by the Boruta algorithm and its iterative process. The horizontal axis represents the input variables, and the vertical axis denotes the feature importance (Z-scores). The blue boxes represent the minimum, average and maximum Z-scores of shadow features, respectively. And the green boxes, yellow boxes, red boxes correspond to confirmed features, tentative features and rejected features, accordingly.

In this study, the Boruta algorithm was used to select the following variables as predictors for the logistic regression model: frailty, cholesterol, WHtR, eGDR, uric acid. The results revealed that a higher eGDR (OR = 0.54, 95% CI: 0.30–0.84) was associated with a reduced prevalence of developing MetS in sarcopenic patients, consistent with the findings from the univariate screening approach; in contrast, frailty (OR = 1.47, 95% CI: 1.01–2.94), elevated cholesterol levels (OR = 3.94, 95% CI: 2.47–6.69), were significantly related to an increased prevalence of MetS, as shown in Figure 2.

**Figure 3:**
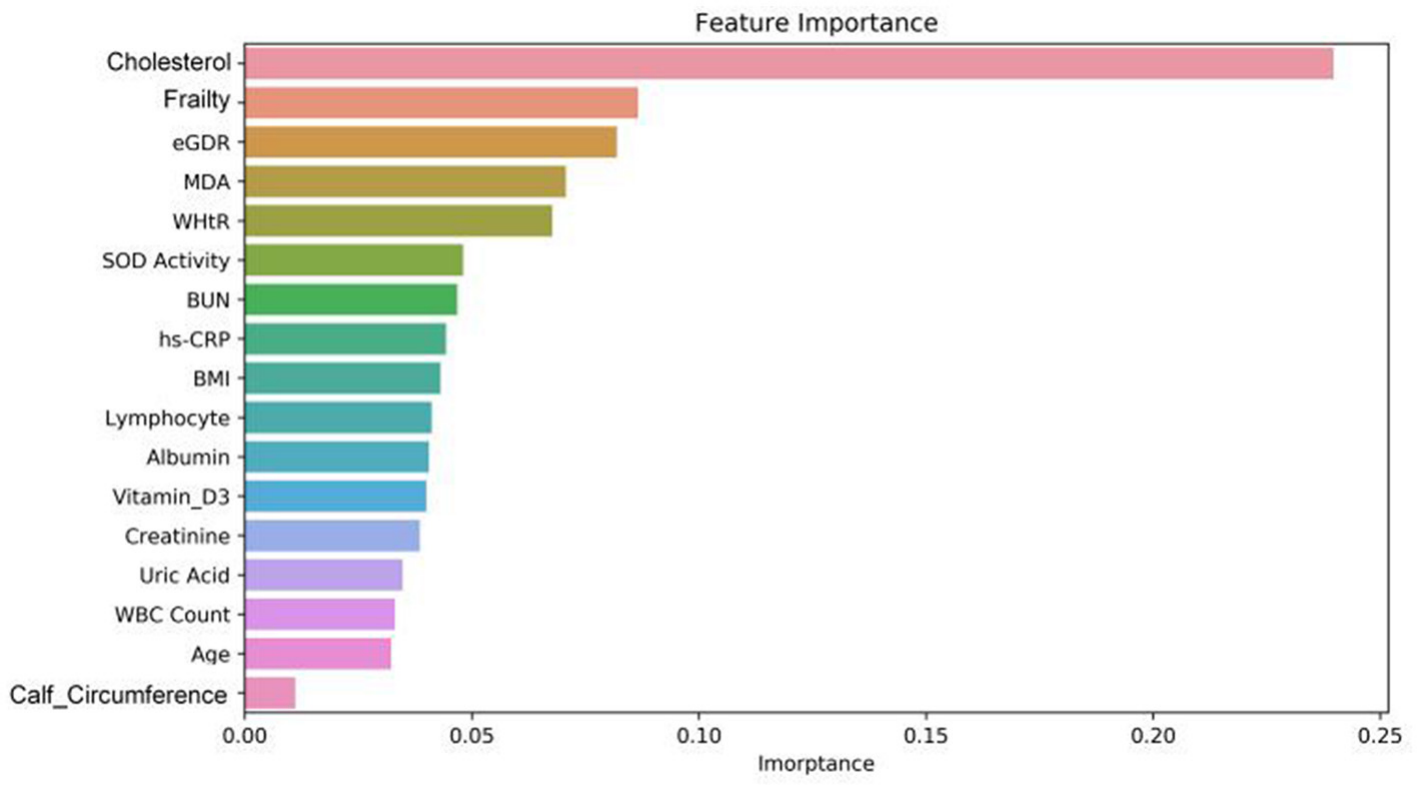
Top 10 Feature Subsets Selected Based on the SVM-RFE Algorithm.

We also used the SVM-RFE algorithm to identify the top 5 feature variables for constructing a regression model, and the ranked list of these variables in descending order of importance is as follows: cholesterol, frailty, eGDR, MDA, WHtR. These findings indicated that a higher eGDR (OR = 0.54, 95% CI: 0.30–0.82) was associated with a reduced prevalence of MetS in sarcopenic patients, consistent with the results from the two previously described models; in contrast, variables significantly related to an increased prevalence of MetS included frailty (OR = 1.63, 95% CI: 1.01–2.72), hihger cholesterol levels (OR = 3.75, 95% CI: 2.39–6.28), aligning with the findings from the Boruta-based algorithm model, as depicted in Figure 3.

**Figure 4:**
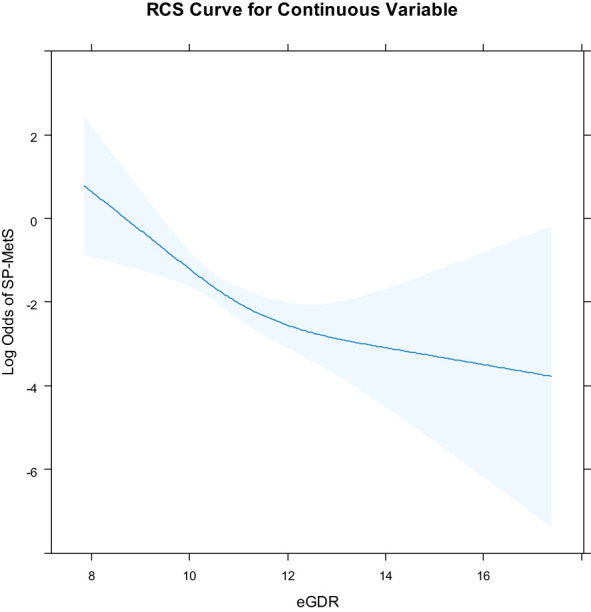
RCS analysis of the association between glucose disposal rate and MetS in elderly individuals with sarcopenia. SP-MetS represents the sarcopenia combined with the MetS group.

Moreover, multicollinearity analyses revealed that the VIF for all variables included in the models was below 5, indicating no significant collinearity among these variables. RCS analysis revealed a significant association between eGDR and the risk of MetS (*P* < 0.01). The non-linear term was not statistically significant (*P* = 0.21), suggesting that a linear relationship adequately describes the correlation, as illustrated in Figure 4.

### 3.4 Internal validation and model performance comparison

Internal validation was performed for all three models using Bootstrap resampling. Results showed that the regression model built by univariate screening yielded a bias-corrected AUC of 0.67, with a average optimism of 0.032 (95% CI: −0.05–0.53). The bias-corrected AUC values of the regression models constructed through Boruta and SVM-RFE algorithms were 0.75 and 0.71 respectively, with average optimism of 0.036 (95% CI: −0.039–0.108) and 0.039 (95% CI:−0.039–0.108) respectively.

In the comparison of model performance, the models that included variables selected by machine learning algorithms showed improved sensitivity and specificity in predicting MetS based on eGDR. The AUCs of the regression models built using variables selected by the Boruta algorithm and the SVM-RFE algorithm were 0.78 and 0.75, respectively, while the AUC of the univariate screening-based model was 0.70. In terms of DeLong's test, the AUC values of the univariate screening model were significantly lower than those of the machine learning-based models (*P* < 0.05). No significant difference in AUC was observed between the two machine learning models (*Z* = 1.28, *P* = 0.60).

### 3.5 Subgroup analysis and sensitivity analysis

**Figure 5:**
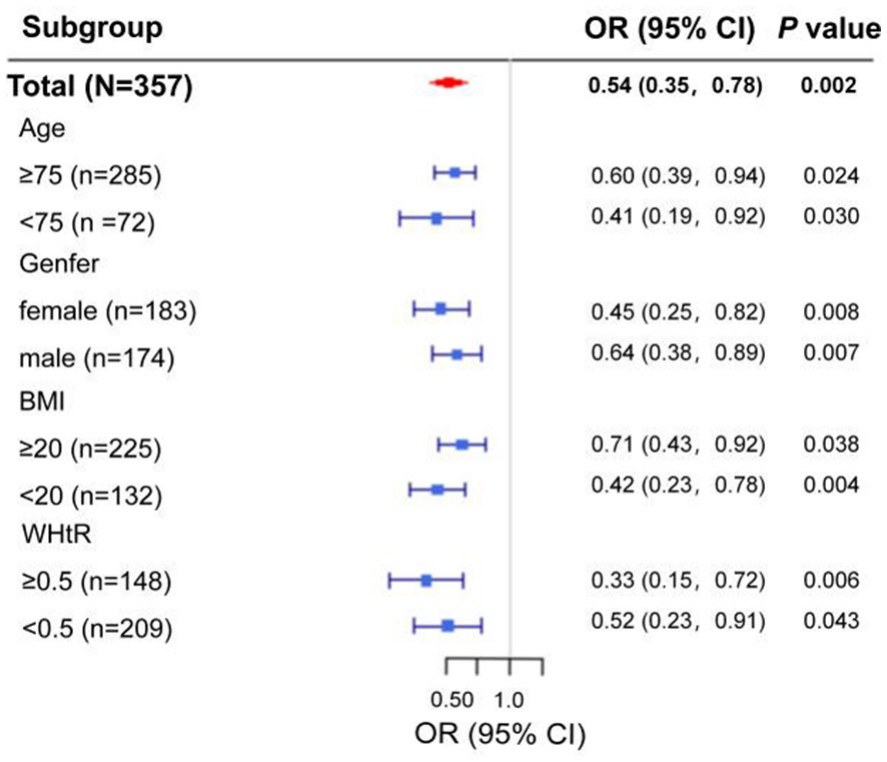
Subgroup analysis of the association between glucose disposal rate and MetS in elderly patients with sarcopenia.

In this study, we also conducted stratified analyses by gender, age, BMI, WHtR, and the results from subgroup analyses indicated that the direction and magnitude of the association between eGDR and MetS in each subgroup were consistent with those observed in the overall analysis (OR = 0.54; 95% CI: 0.35–0.78), suggesting the robustness of this association, as illustrated in Figure 5. LOO analysis demonstrated that eGDR still remained associated with metabolic syndrome after sequential omission of either waist circumference or blood pressure—yielding adjusted odds ratios (aORs) of 0.63 (95% CI: 0.27–0.95) and 0.75 (95% CI: 0.16–0.98), respectively.

## 4 Discussion

With the accelerating aging population in China, the disease burden imposed by chronic degenerative conditions on public health has attracted increasing attention (25). Effectively preventing, treating, and managing age-related diseases to promote healthy aging has become a critical public health challenge. Sarcopenia, a prevalent condition among older adults, is marked by the loss of lean body mass and deterioration of musculoskeletal function (26). Among the 970 older adults included in this study, the prevalence of sarcopenia was 36.80% (357/970), which is higher than the reported overall prevalence of 16.5% among community-dwelling older adults in China (27). This suggests that hospitalized older adults are at particularly high risk for sarcopenia. A meta-analysis on the prevalence of sarcopenia among older adults in China indicated that the overall prevalence among hospitalized older patients ranges between 30% and 40% (3), aligning with the findings of this study. It is important to note that sarcopenia is not an independent disease; rather, the loss and reduction of muscle mass often co-occur with other conditions like IR and dyslipidemia, which are manifestations of metabolic disorders. In this study, it was found that the prevalence of MetS in sarcopenic patients was relatively high (14.01%, 50/357), significantly higher than that in the non-sarcopenic group (3.10%, 19/613). This finding is consistent with previous multi-regional studies (28–30), indicating a significant association between sarcopenia and MetS. Owing to factors such as chronic inflammation, visceral fat accumulation, as well as IR, patients with both sarcopenia and MetS frequently face a higher risk of adverse health events, particularly an increased incidence of frailty and mortality (31). In this study, among the 357 sarcopenic patients, the prevalence of frailty in those with MetS was 66% (33/50), which was significantly higher than that in patients with sarcopenia alone (50.81%, 156/307), with the difference being statistically significant. This finding further supports the notion that sarcopenia combined with MetS is related to an increased risk of frailty.

Skeletal muscle serves as the key organ for insulin-stimulated glucose uptake and utilization, and its functional status directly influences the whole-body eGDR level during insulin stimulation (32, 33). eGDR is a highly clinically significant composite indicator, mainly utilized to evaluate IR. It can also simultaneously reflect the cumulative burden of metabolic risk factors such as hypertension and dyslipidemia. In this study, we focused on measuring eGDR and exploring its relationship with MetS in older adults with sarcopenia, highlighting the importance of skeletal muscle metabolic capacity, especially when sarcopenia coexists with metabolic comorbidities (34). In this research, the median eGDR of sarcopenia subjects was 10.82 mg/kg/min, while in patients suffering from both sarcopenia and MetS, it dropped down to 9.60 mg/kg/min of eGDR. This observation suggests that poor skeletal muscle insulin sensitivity might not only represent the pathophysiological substrate of sarcopenia in combination with MetS, but also a key mechanism that synergistically exacerbates IR and frailty (35).

In addition to the difference in eGDR, the sarcopenia with MetS group exhibited slightly higher serum albumin and total cholesterol levels compared to the sarcopenia group. Notably, albumin is a negative acute-phase protein. Its hepatic synthesis is suppressed during active systemic inflammation or acute physiological stress, resulting in reduced circulating concentrations (36). In this study, patients with sarcopenia and MetS exhibited a significantly higher serum albumin concentration (43.96 ± 3.08 g/L) compared with those with sarcopenia (42.51 ± 3.96 g/L). The elevated albumin levels observed in the sarcopenia with MetS group may reflect potential differences in nutritional reserves and liver synthetic capacity (37). In addition, the use of diuretics and statins may also lead to a higher albumin level in the sarcopenia with MetS group compared to the sarcopenia group (38). Secondly, dyslipidemia is a core pathological feature of MetS, and elevated total cholesterol—though not part of the formal diagnostic criteria—is a frequently observed clinical finding. Previous studies have consistently reported dyslipidemia in patients with MetS, including elevated LDL-C and abnormalities in other lipid parameters (39). In this study, the sarcopenia with MetS group had a total cholesterol level of 5.36 ± 1.02 mmol/L—significantly higher than that of patients with sarcopenia alone (4.63 ± 0.92 mmol/L; *P* < 0.01)—in agreement with prior reports (40).

As for inflammation and oxidative stress, the levels of MDA, uric acid levels, white blood cell count, and lymphocyte count in patients with sarcopenia and MetS were higher than those in sarcopenia group. In this study, the malondialdehyde level in patients with sarcopenia combined with MetS was 9.16 ± 8.06 μmol/L; while in patients with sarcopenia alone, it was 6.97 ± 3.88 μmol/L. As a final product of lipid peroxidation, elevated MDA levels typically signal an increased oxidative state, and this increase may stem from excessive production of mitochondrial reactive oxygen species (ROS) due to elevated free fatty acids (FFAs), as well as reduced glutathione (GSH) levels and increased oxidized glutathione (GSSG) resulting from the loss of skeletal muscle mass (41, 42). White blood cell and lymphocyte counts are commonly used clinical indicators for evaluating systemic inflammation, and their elevation often indicates the presence of an ongoing inflammatory process. In this study, the white blood cell and lymphocyte counts in the sarcopenia with MetS group were 6.45 × 109/L and 2.23 × 109/L respectively, which were higher than those in the sarcopenia group and the differences were statistically significant. IR can impair renal uric acid excretion, while increased oxidative stress accelerates ATP catabolism and purine turnover, boosting uric acid production. This partially accounts for the higher uric acid levels in the sarcopenia with MetS group (341.94 μmol/L) compared to the sarcopenia group (290.27 μmol/L) (43). These results suggest that compared with sarcopenia patients, sarcopenia patients with MetS may have higher levels of inflammation and oxidative stress. However, this study is a cross-sectional study, which cannot effectively control the potential impact of confounding bias on the research results. Studies should adopt prospective research methods such as cohort studies to further explore the correlation in future.

The analysis of the relationship between eGDR and MetS in sarcopenic patients revealed that, regardless of how the logistic regression model was constructed, eGDR was negatively associated with MetS, aligning with previous research (44). After screening variables using the Boruta algorithm and constructing a regression model based on the selected variables, five characteristic variables were ultimately identified: frailty, cholesterol, WHtR, eGDR, uric acid. The OR for eGDR was 0.54 (95% CI: 0.30~0.84), indicating that a lower eGDR level is linked to an increased prevalence of MetS in elderly individuals with sarcopenia. Rubio-Ruiz et al. (45) demonstrated that, as the primary site for glucose uptake and utilization, skeletal muscle dysfunction often presents as abnormal IR markers, which in turn contribute to the development of MetS and various adverse health outcomes. In individuals with sarcopenia, a low eGDR may signal an exacerbation of the metabolic disorder process. In addition to low eGDR, the regression equation also identified other possible risk factors for MetS in the sarcopenic population: frailty, elevated cholesterol, higher BMI, uric acid, and MDA. The risk of MetS in patients with both frailty and sarcopenia was 1.63 times higher than that in individuals without frailty (95% CI: 1.01–2.72), showing that the multisystem functional impairment and disruption of physiological homeostasis associated with frailty may share common pathological pathways with MetS (46).

To assess how different variable selection methods impact prediction accuracy, three variable selection methods were used to construct logistic regression models. We compared the performance of models constructed using variables selected through univariate analysis screening, the SVM-RFE algorithm, and the random forest-based Boruta algorithm. The results showed that, compared with univariate screening, both the SVM-RFE algorithm and the Boruta algorithm produced models with higher predictive performance. The AUC of the regression model based on the SVM-RFE algorithm was 0.75, and that of the model based on the Boruta algorithm was 0.78. In contrast, the regression model based on univariate screening had an AUC of only 0.70, which was significantly lower than that of the models built using the two machine learning algorithms (*P* < 0.05). This suggests that the conventional univariate screening approach may not adequately account for interactions and non-linear relationships between variables, potentially leading to reduced generalization ability due to the omission of important predictors or the inclusion of noise variables; in contrast, the SVM-RFE and Boruta algorithms may be more effective in selecting features that capture synergistic effects among variables or non-linear relationships with the outcome, thus enabling the construction of more discriminative and clinically useful prediction models.

## Data Availability

The original contributions presented in the study are included in the article/supplementary material, further inquiries can be directed to the corresponding authors.
